# Weather Variability, Tides, and Barmah Forest Virus Disease in the Gladstone Region, Australia

**DOI:** 10.1289/ehp.8568

**Published:** 2005-12-15

**Authors:** Suchithra Naish, Wenbiao Hu, Neville Nicholls, John S. Mackenzie, Anthony J. McMichael, Pat Dale, Shilu Tong

**Affiliations:** 1 School of Public Health, Queensland University of Technology, Queensland, Australia; 2 Bureau of Meteorology Research Centre, Melbourne, Australia; 3 Department of Microbiology and Parasitology, University of Queensland, Queensland, Australia; 4 National Centre for Epidemiology and Population Health, Australian National University, Canberra, Australia; 5 Australian School of Environmental Studies, Griffith University, Nathan, Queensland, Australia

**Keywords:** Barmah Forest virus, control, forecasting, Gladstone region, risk factors, time series modeling

## Abstract

In this study we examined the impact of weather variability and tides on the transmission of Barmah Forest virus (BFV) disease and developed a weather-based forecasting model for BFV disease in the Gladstone region, Australia. We used seasonal autoregressive integrated moving-average (SARIMA) models to determine the contribution of weather variables to BFV transmission after the time-series data of response and explanatory variables were made stationary through seasonal differencing. We obtained data on the monthly counts of BFV cases, weather variables (e.g., mean minimum and maximum temperature, total rainfall, and mean relative humidity), high and low tides, and the population size in the Gladstone region between January 1992 and December 2001 from the Queensland Department of Health, Australian Bureau of Meteorology, Queensland Department of Transport, and Australian Bureau of Statistics, respectively. The SARIMA model shows that the 5-month moving average of minimum temperature (β = 0.15, *p*-value < 0.001) was statistically significantly and positively associated with BFV disease, whereas high tide in the current month (β = −1.03, *p*-value = 0.04) was statistically significantly and inversely associated with it. However, no significant association was found for other variables. These results may be applied to forecast the occurrence of BFV disease and to use public health resources in BFV control and prevention.

Barmah Forest virus (BFV) disease is one of the common human arboviral (arthropod-borne) diseases causing public health concerns in Australia (Russell and Dwyer 2000; Russell and Kay 2004). BFV is a nonfatal disease but causes a syndrome similar to that of Ross River virus disease (Flexman et al. 1998; Mackenzie et al. 1998; Sam and Crerar 1996), characterized by arthralgia, myalgia, fever, and rash (Beard et al. 1997; Boughton et al. 1988; Dore and Auld 2004; Flexman et al. 1998; Mackenzie and Smith 1996; Nash and Harrington 1991; Phillips et al. 1990; Sam and Crerar 1996). Other uncommon symptoms are glomerulonephritis and Guillain-Barre syndrome, which includes kidney inflammation (Katz et al. 1997; Lucas and Qiao 1999; Phan et al. 1998). Sometimes the symptoms persist for up to several months (Lindsay et al. 1995a ) and may lead to chronic illness (Sam and Crerar 1996). The intrinsic incubation period of the disease is about 7–9 days (Mackenzie and Smith 1996).

BFV was named after it was first isolated from *Culex annulirostris* mosquitoes trapped in 1974 from the Barmah Forest area of the Murray River in northern Victoria, Australia (Marshall et al. 1982). However, its association with human “infection” was not identified until 1986 (Vale et al. 1986), and it was not associated with human “disease” until 1988 (Boughton et al. 1988; Mackenzie et al. 1994). The first recognized epidemic of BFV disease occurred at Nhulunbuy in the Northern Territory in 1992 (Merianos et al. 1992). Most BFV cases occur during or just after outbreaks of Ross River virus disease, suggesting that BFV and Ross River viruses may have similar mosquito vectors and require similar environmental conditions for successful transmission (Lindsay et al. 1995b).

BFV has been isolated from > 73 species of mosquitoes belonging to four genera: *Aedes*, *Culex* (some *Aedes* species were renamed *Ochlerotatus*) (Reinert 2000), *Anopheles*, and *Coquillettidia* (Boyd et al. 2001; Doggett and Russell 1997; McMichael 2003; Russell 1995; Watson and Kay 1999). Previous studies show that marsupials (e.g., kangaroos and wallabies) are possible (reservoir) hosts for BFV (Doherty et al. 1971; Jeffery et al. 2002; Kay and Aaskov 1989; Sam and Crerar 1996; Vale et al. 1991; van Buynder et al. 1995). Some studies suggest that birds may also play a major role in the transmission of this disease (Kay and Aaskov 1989). However, brushtail possums, cats, and dogs are unlikely to be important hosts for BFV in Australia (Boyd et al. 2001; Boyd and Kay 2002; Watson and Kay 1999).

BFV cases have been reported in every state across Australia, particularly in tropical and subtropical regions (Lindsay et al. 1995b; Mackenzie et al. 1994; Merianos et al. 1992; Poidinger et al. 1997; Russell 1998a). For example, in 2002 the rate of notification (10.5/100,000) in Queensland was much higher than the national average (3.2/100,000).

For the transmission of BFV, the virus and its reservoir, the vector, the human population, and environmental conditions are key factors. The virus depends on the continuing presence of nonimmune hosts in the reservoir population. Weather conditions directly affect the breeding, survival (Reeves et al. 1994), and abundance of mosquitoes and their extrinsic incubation period (Hardy 1988; Kramer et al. 1983; Turell 1993). However, other socioecologic factors such as vector, virus, human behavior, lifestyles, and herd immunity may also be involved in the transmission of BFV disease. Mosquito abundance is an important factor for the distribution of BFV disease, and studies show that climatic factors such as temperature and rainfall have significant impacts on mosquito populations (Lindsay and Mackenzie 1998; Russell 1998a) at local and regional levels. The breeding habits and survival of mosquitoes depend largely on weather variability (e.g., temperature and rainfall) and tides (Weinstein 1997).

However, few empirical studies have examined the environmental predictors for the transmission of BFV. Hence, in this study we aimed to assess the impact of weather and tidal variability on the transmission of BFV disease in the Gladstone region of Australia and to develop a weather-based epidemic-forecasting model for the control and prevention of BFV disease.

## Materials and Methods

### Study area.

**Figure 1:**
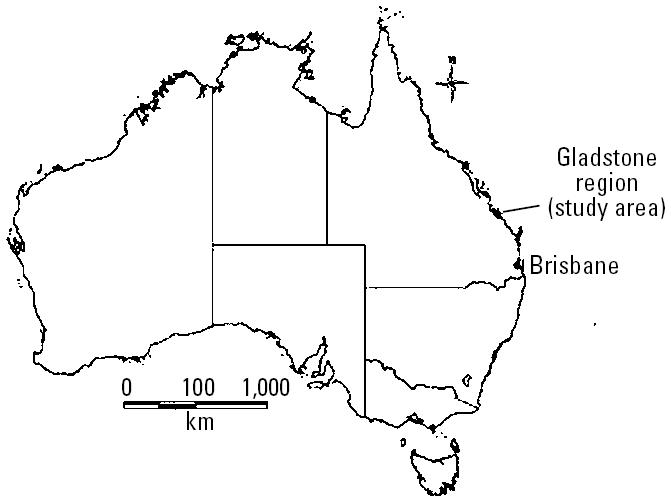
Map showing the location of the study area in Australia.

Gladstone is a subtropical area situated on the east coast of Australia 550 km north of Brisbane, the capital of the State of Queensland (Figure 1). The Gladstone region was chosen as the study area because of its relatively high incidence of BFV between 1992 and 2001 (average annual incidence, 34.8/100,000). According to Australian Bureau of Statistics (2004), the population size in Gladstone region was 182,169 on 30 June 2001.

### Data collection.

We obtained computerized data sets of notifications of BFV cases in the Gladstone region for the period 1 January 1992 through 31 December 2001 from the Queensland Department of Health. The communicable diseases notification system is operated under the guidance of the Communicable Diseases Network Australia (Queensland Department of Health 2004). Monthly incidence rates of BFV were used in this study. Weather data of daily maximum and minimum temperature, rainfall, and relative humidity were supplied by the Australian Bureau of Meteorology (2004). We aggregated the daily data and obtained average monthly data for maximum and minimum temperature, relative humidity, and rainfall. Semidiurnal data on daily high and low tides along the Gladstone coast were provided by the Queensland Department of Transport (2004) because tides have been identified as a risk factor for mosquito-borne diseases in Queensland (Tong and Hu 2001; Tong et al. 2004). Tides are the vertical rising and lowering of sea level. Usually two high and two low tides occur within each 25 hr and 50 min. We aggregated the high and low tides and obtained average monthly high and low tide data. Population data for the study period were obtained from the Australian Bureau of Statistics (2004).

### Data analysis.

We conducted univariate analyses for each independent variable. We used cross-correlations to assess associations between weather and low- and high-tide variables and the incidence of BFV disease over a range of lags (Chatfield 1975). We used the multivariate seasonal autoregressive integrated moving-average (SARIMA) model to examine the independent contribution of weather and tidal variables to BFV transmission. Box-Jenkins (time-series) modeling strategy was useful in constructing SARIMA models for vector-borne diseases because it is a powerful tool for interpreting and applying surveillance data in disease control, prevention, and forecast; it has the capacity to analyze a long series of data in a stationary mode (Allard 1998).

Before analysis, we calculated monthly incidences of BFV disease using monthly counts of BFV as a numerator and population size in the middle of each year as a denominator. To reduce the impact of extreme values (e.g., outbreaks), we applied a natural logarithmic (ln) transformation for the incidence of BFV disease (Box et al. 1994). To achieve appropriate stationary time series, all dependent and independent variables were seasonally differenced with regard to yearly periodicity. Because zero values (no cases of BFV) were present in some months, log(BFV incidence + 1) was used in transformation. However, these values were transformed back to zero in the final stage of analysis.

The modeling of the relationship between the variables and BFV transmission involved three stages: identification, estimation, and diagnosis. In the identification stage, we determined the need for differencing the monthly BFV incidences by checking stationarity (i.e., trends in the mean and variance) and the order of both the seasonal and non-seasonal autoregressive and moving-average indicators by using an autocorrelation function and a partial autocorrelation function (Hipel et al. 1977).

In the estimation stage, we developed SARIMA models using the log-transformed monthly incidence of BFV as a dependent variable and seasonally differenced monthly weather variables and tidal height as explanatory variables. The following were the parameters selected when fitting the SARIMA model: *p*, the order of autoregression; *d*, the degree of difference; *q*, the order of moving average; *P*, the seasonal autoregression; *D*, the seasonal integration and *Q*, the seasonal moving average. Hence, the model used in this study was SARIMA (*p*,*d*,*q*)(*P*,*D*,*Q*)*_s_* (where *s* is the length of the seasonal period). We used the stepwise SARIMA method to select the weather and tidal height variables. Our main criterion for judging a model against other models was based on the lowest value in Akaike’s Information Criterion (AIC) (Akaike 1974).

In the diagnostic stage, the goodness of fit of the models was determined for appropriate modeling, using both time series (e.g., autocorrelation function and partial autocorrelation function of residuals) and classic tools (e.g., checking the normality of the residuals) (Tabachnick and Fidell 2001).

Finally, we applied the model to forecast the transmission of BFV disease. There were two major steps involved in this procedure. First, the main data file was divided into two data sets: the data between January 1992 and December 2000 were used to construct the SARIMA model, and the data between January and December 2001 were used to validate the model. We chose the 2001 year as representative year because SARIMA is a powerful tool to predict future situation (i.e., 2001 in this case) using continuous historic data (i.e., 1992–2000). However, we also validated the model using either 2-year (i.e., 2000–2001) or 3-year data (i.e., 1999–2001) and obtained similar patterns (further data are available from the corresponding author). Additionally, we used cross-correlation coefficients to investigate agreement between actual and predicted values at various lags. A plot of cumulative sums for actual and predicted values was also used to show the model agreement. Finally, we evaluated the predictive validity of this model by using the root mean square (RMS) error and RMS percentage error criterion (Makridakes et al. 1998). The smaller the RMS error, the better the model for forecasting. All these analyses were undertaken using SPSS for Windows (Statistical Package for Social Sciences 2004).

## Results

### Descriptive analyses.

Descriptive statistics on incidence of BFV and weather variables and tides for the Gladstone region for the period 1992–2001 are presented in Table 1. There was a considerable variation for each of these variables.

The results of the cross-correlations show that the incidence of BFV disease was statistically significantly associated with minimum temperature at the current month and at lags of 2–5 months, maximum temperature at lags of 3–4 months, low tide at the current month and at lags of 1 and 5 months, and high tide at the current month. A similar pattern was observed for the actual and predicted incidence rates of BFV (Table 2). However, there was no association of BFV with rainfall and relative humidity.

Correlations between the independent variables (Table 3) indicate that maximum and minimum temperatures were highly correlated with each other (*r_s_* = 0.95), whereas other correlations were not strong (|*r_s_*| ≤0.57). Therefore, maximum and minimum temperatures were included separately in the models to avoid muliticollinearity.

**Figure 2:**
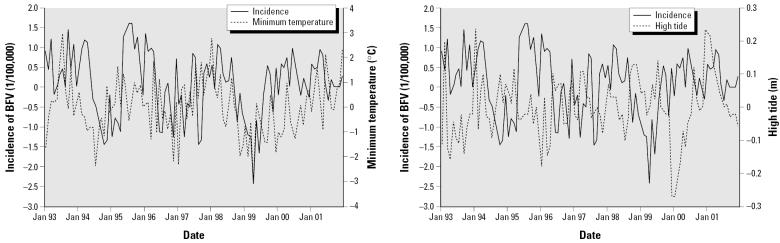
Relationships between monthly incidence of BFV disease and minimum temperature (*A*) and high tide (*B*) using seasonal differencing in the Gladstone region during the period 1992–2001.

To ensure that the time series of weather variables and the incidence of BFV disease were stationary, we calculated seasonally differenced values. Figure 2 shows that seasonally differenced weather variables appeared to be associated with the incidence of BFV disease. However, a BFV outbreak during 1995–1996 did not seem to match up well with individual variables, possibly because of the multiple factorial nature of an outbreak and the lagged effects of weather variability.

### SARIMA models.

We calculated a series of models using a range of independent variables including population size, minimum and maximum temperature, rainfall, relative humidity, and high and low tide variables. A backward elimination method was adopted to select the most suitable model, based on the AIC. Of the models tested, a SARIMA model [(1,0,0)(1,0,1)_12_] with a moving average of 0-, 2-, 3-, 4-, and 5-month lags for minimum temperature and high tide in the current month was the best fit for this data set (AIC value, 168.7) (Table 4).

The results of the SARIMA model [(1,0,0)(1,0,1)_12_] show that autoregression (β = 0.52, *p*-value < 0.0001), seasonal autoregression (β = 0.81, *p*-value < 0.0001), seasonal moving average (β = 0.68, *p*-value = 0.01), monthly minimum temperature with a moving average of 0-, 2-, 3-, 4-, and 5-month lags (β = 0.15, *p*-value < 0.0001), and high tide at the current month (β = −1.03, *p*-value = 0.04) were statistically significantly associated with the incidence of BFV disease. However, maximum temperature, rainfall, relative humidity, and low tide were not statistically significantly associated with the incidence of BFV disease at any lags after adjustment for autocorrelation, seasonality, and other covariates.

**Figure 3:**
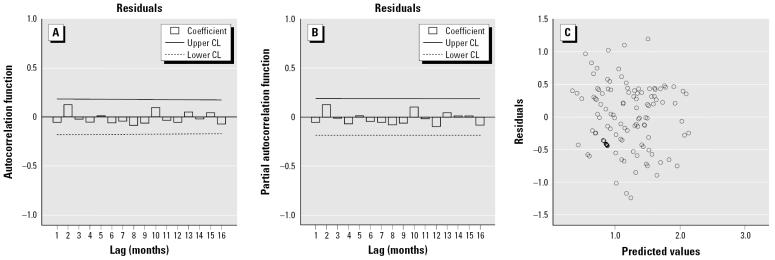
Diagnostic checking. (*A*) Simple autocorrelation function. (*B*) Partial autocorrelation function. (*C*) Scatter plot of residuals from the seasonal autoregressive, integrated, and moving-average (SARIMA) fitting model. CL, confidence limit.

The goodness-of-fit analyses show that there was no significant autocorrelation between residuals at different lags in the SARIMA model (Figure 3), and the model fitted the data reasonably well.

### Validation model.

**Figure 4:**
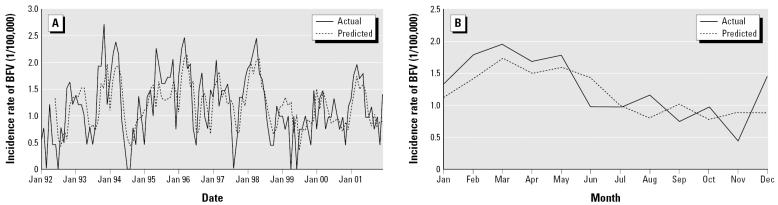
(*A*) SARIMA model of forecasting weather variation in Gladstone region. (*B*) Validation model for the period 1 January through 31 December 2001 with the incidence of BFV (1/100,000).

**Figure 5:**
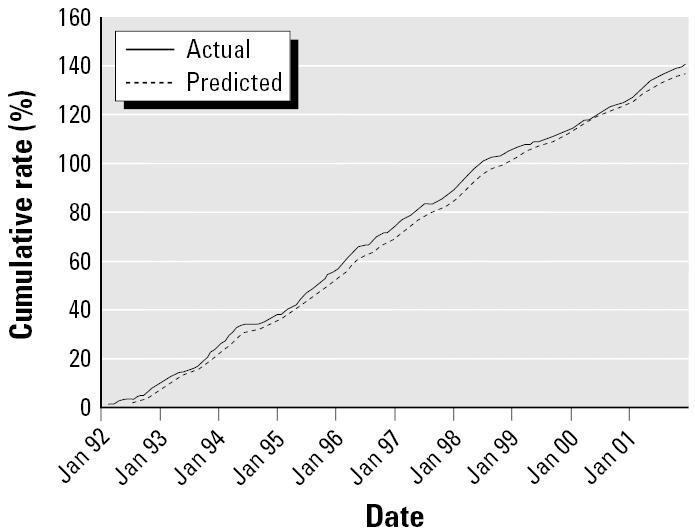
Time series plot of cumulative sums for actual and predicted values of BFV incidence.

The model developed using the data between January 1992 and December 2000 was applied to predict the transmission of BFV and was then validated using the data between January and December 2001. The results of the validation analyses show that the parsimonious model SARIMA (1,0,0)(1,0,1)_12_ was an appropriate model for forecasting the epidemics of BFV disease in Gladstone region because the RMS error was small (1.2; RMS percentage error = 0.73%). In addition, Figure 4 shows that the predictive, and actual values matched reasonably well and there was a consistency in the trend (Figure 4). To validate these results, a plot of time series on the cumulative sums for actual and predicted incidence values is presented in Figure 5.

## Discussion

Recently, an analysis of spatiotemporal patterns of BFV disease in Queensland indicated that the geographic distribution has been expanding over the last decade (Tong et al. 2005). However, the reasons for its geographic expansion remain largely unknown. In this study, we applied a multivariate SARIMA model to assess the independent effects of weather variables (minimum and maximum temperature, rainfall, and relative humidity) and high and low tides on the transmission of BFV disease in Gladstone region because this model has been increasingly used for research on vector-borne and other infectious diseases (Allard 1998; Helfenstein 1991; Stroup et al. 1988; Tong and Hu 2001). The results of this study suggest that weather variability, particularly minimum temperature and high tide, play a significant role in the transmission of BFV disease in Gladstone region of Australia. These results are consistent with other vector-borne disease research (Lindsay and Mackenzie 1998; Tong and Hu 2001; Tong et al. 2004; Weinstein 1997).

Previous research indicates that mosquitoes that transmit BFV are sensitive to temperature (Russell 1998b). However, it remains unclear which of the weather parameters (e.g., maximum or minimum temperature) is important in the determination of BFV transmission. Our results suggest that minimum rather than maximum temperature plays a significant role in the BFV transmission cycles in the Gladstone region. Temperature influences the length and efficiency of incubation periods of mosquitoes and the survival of adult mosquitoes (McMichael 2003; Reeves et al. 1994; Russell 1995). Higher temperatures increase the rate of larval development, with adults emerging faster than at lower temperatures, and thus may increase the likelihood of BFV transmission. For example, some species of mosquitoes are temperature specific in their breeding (Hardy 1988; Mackenzie et al. 1994; Russell 1995).

In this study, however, we found that minimum temperature with lags of 0, 2, 3, 4, and 5 months is an important determinant of BFV incidence. The reason for this is largely unknown. However, this may be because Gladstone region has subtropical weather, and the dominant species of mosquitoes in this region may be sensitive to minimum temperature rather than maximum temperature. Minimum temperature not only influenced the disease in the current month but also had time lag effects. Lags of 2–5 months were detected in this study. This lag effect may reflect the life course for the development of mosquitoes, intrinsic and extrinsic incubation periods, and cyclic changes in both the vector (e.g., biting pattern) and human behavior (e.g., outdoor activity) (Hu et al. 2004).

We also found that high tide at the current month was negatively associated with BFV transmission. Rapid fluctuations in water level typically reduce mosquito breeding sites because mosquito larvae live at or immediately below surface water and breathe oxygen (Collins and Resh 1989). If the surface water is disturbed, mosquito larvae may drown. In addition, cyclones often occurred in the Gladstone region (Queensland Department of Transport 2004). Gladstone sometimes faces king tides (those well above average; average height of king tides was 4.7 m for the period 1992–2001). Thus, the mosquito breeding sites may be flushed out by very high tides. Clearly, a combination of one or more of these factors might have decreased the incidence of BFV disease in Gladstone. Therefore, further studies on the ecology of BFV transmission are required to elucidate the underlying reasons for this finding.

The strengths of this study are that *a*) to our knowledge, this is the first study to look at the response of BFV disease to weather variability and tides; *b*) time-series models were conducted using a range of weather and tidal variables, and various sources of data were used; *c*) residual analyses show that the SARIMA model fitted the data reasonably well; and *d*) the model developed in this study had a good accuracy for forecasting BFV disease.

The limitations of this study are that, first, the quality of data in the national disease surveillance system may vary with time and place. For example, the awareness of BFV disease might have increased among medical practitioners and the general public over the last decade, and it may be one of the reasons for the apparent increased trend of this disease. However, the quality of data is unlikely to change dramatically at the temporal scale used in this study (i.e., monthly). Second, the ecology of BFV is quite complex. There are many factors such as virus, vector, host, and environment that are involved in the transmission cycles of BFV. Weather variation; virus strain; mosquito densities, survival, and breeding; human activities and movement; socioeconomic status; and population immunity may all contribute to the transmission cycles of BFV. Changes in agricultural practice—such as building dams and irrigation systems—have created ideal larval habitats for selected specie of mosquitoes. Clearing forests for agricultural use and urban development (near wetland) could increase the potential for BFV transmission (Lindsay and Mackenzie 1998; Mackenzie et al. 2000; Tong and Hu 2002). The increased human populations living in intimate contact with increasingly high densities of mosquito populations (i.e., around wetland and salt-marsh habitats) create ideal conditions for increased BFV. Tourism and travel have also become important mechanisms for facilitating the BFV and its vectors. Periodic changes of climate may also influence the local weather conditions and the life cycle of the disease reservoirs and cyclic changes in human activities (e.g., outdoor activities). However, data were unavailable on many of these factors, and the possibility of some “hidden effects” of weather on the results cannot be entirely ruled out. Finally, it was difficult to generalize the findings of this study to other areas as only local data were used.

In conclusion, the results of this study show that minimum temperature and high tide were the key weather determinants of BFV disease transmission in Gladstone region. The development of epidemic-forecasting systems is important in the control and prevention of infectious disease. Should an outbreak of BFV occur, a large-scale public health intervention is usually required. Early warning systems based on weather forecasts can assist in improving vector control and personal protection. The results of this type of analysis provide an opportunity to develop early warning and improve vector control, community intervention, and personal protection. Forecasted weather change (particularly in minimum temperature) may increase mosquito activity and transmission of BFV disease in the area (Jeffery et al. 2002; Kelly-Hope et al. 2002). Based on the midrange estimates of future temperature change by the year 2030 of 0.3–2.0°C (Walsh et al. 2002), the incidence of BFV disease is estimated to increase by 0.6–3.9/100,000, if other socioecologic factors remain constant. Therefore, public health authorities need to be prepared for a likely increase in transmission of BFV disease in the Gladstone region. However, the global warming trend is likely to affect other socioecologic factors. For example, people may undertake more outdoor activities and would then be more likely to be bitten by mosquitoes, as temperature increases. Therefore, our health risk assessment for the projected climate change may be conservative. Nevertheless, our attempt to forecast epidemics of BFV should be an impetus for future studies on vector control and public health interventions on the transmission of BFV disease, particularly in high-risk areas.

## Figures and Tables

**Table 1:** Descriptive analyses for the weather and tidal variables at Gladstone region during the period 1992–2001.

Variable	No.	Mean ± SD	Minimum	Maximum
Incidence of BFV				
Overall	120	2.91 ± 2.56	0.00	13.90
Summer	30	3.22 ± 2.04	0.56	8.32
Autumn	30	3.98 ± 3.14	0.00	10.54
Winter	30	1.73 ± 1.49	0.00	5.63
Spring	30	2.70 ± 2.81	0.55	13.90
Maximum temperature (°C)	120	27.67 ± 3.07	21.87	33.40
Minimum temperature (°C)	120	18.75 ± 3.23	12.44	24.27
Precipitation (mm)	120	56.91 ± 64.01	0.40	498.20
Relative humidity (%)	120	92.08 ± 9.18	62.00	100
High tide (m)	120	1.57 ± 0.07	1.42	1.81
Low tide (m)	120	−1.36 ± 0.09	−1.58	−1.12

**Table 2:** Cross-correlation coefficients of actual and predicted incidence rates of BFV and weather variability in Gladstone.

Variable	Lag 0	Lag 1	Lag 2	Lag 3	Lag 4	Lag 5
Maximum temperature						
Actual	0.037	0.105	0.183	0.301*	0.225*	0.152
Predicted	0.021	0.077	0.183	0.205	0.333*	0.234*
Minimum temperature						
Actual	0.216*	0.152	0.385*	0.346*	0.411*	0.228*
Predicted	0.267*	0.178	0.254*	0.426*	0.368*	0.458*
Rainfall						
Actual	−0.020	−0.166	−0.081	−0.112	−0.074	−0.129
Predicted	0.107	−0.078	−0.079	−0.125	−0.133	−0.005
Relative humidity at 0900 hr						
Actual	0.022	−0.001	−0.057	−0.093	0.063	−0.152
Predicted	0.243*	0.178	0.064	0.115	−0.025	0.146
High tide						
Actual	−0.238*	−0.131	−0.110	−0.103	−0.145	−0.180
Predicted	−0.336*	−0.231*	−0.169	−0.118	−0.092	−0.144
Low tide						
Actual	−0.368*	−0.313*	−0.163	−0.087	−0.197	−0.240*
Predicted	−0.342*	−0.339*	−0.281*	−0.176	−0.066	−0.224

* *p*-Value < 0.05.

**Table 3:** Intercorrelations between independent variables.

Variable	Maximum temperature	Relative humidity 0900 hr	Rainfall	High tide	Low tide
Minimum temperature	0.95**	−0.10	0.40**	0.34**	0.57**
Maximum temperature		−0.22**	0.30**	0.31**	0.52**
Relative humidity 0900 hr			0.18*	−0.12	−0.12
Rainfall				0.05	0.20*
High tide					0.55**

* Correlation is significant at the 0.05 level.

** Correlation is significant at the 0.01 level.

**Table 4:** Regression coefficients of the SARIMA on the monthly incidence of BFV disease in Gladstone region, 1992–2001.

	Model without weather variables^a^			Model with weather variables^b^		
Variables	β	SE	*p*-Value	β	SE	*p*-Value
Autoregression	0.76	0.06	0.00	0.52	0.08	0.00
Seasonal autoregression	0.87	0.10	0.00	0.81	0.22	0.00
Seasonal moving average	0.64	0.16	0.00	0.68	0.28	0.01
Minimum temperature	—	—	—	0.15	0.04	0.00
High tide	—	—	—	−1.03	0.51	0.04

—, Variables not included.

a Log likelihood = −98.78; AIC = 203.57 [(1,0,0)(1,0,1)_12_].

b Log likelihood = −79.33, AIC = 168.66 [(1,0,0)(1,0,1)_12_] (best-fit model).
